# Experimental data of the static behavior of reinforced concrete beams at room and low temperature

**DOI:** 10.1016/j.dib.2016.04.025

**Published:** 2016-04-16

**Authors:** M. Mehdi Mirzazadeh, Martin Noël, Mark F. Green

**Affiliations:** a Department of Civil Engineering, Queen’s University, 58 University Ave., Kingston, Ontario, K7L 3N6, USA; b Department of Civil Engineering, University of Ottawa 161 Louis-Pasteur Pvt., Ottawa, Ontario, K1N 6N5, USA

**Keywords:** Concrete bridge, Reinforced concrete, Static behavior, Low temperature, Crack width, DIC/PIV technique, DIC error calibration

## Abstract

This article provides data on the static behavior of reinforced concrete at room and low temperature including, strength, ductility, and crack widths of the reinforced concrete. The experimental data on the application of digital image correlation (DIC) or particle image velocimetry (PIV) in measuring crack widths and the accuracy and precision of DIC/PIV method with temperature variations when is used for measuring strains is provided as well.

## Specifications table

**Table:** 

Subject area	*Civil Engineering*
More specific subject area	*Structural Engineering*
Type of data	*Table, image, text file, graph, figure*
How data was acquired	*Lab Experiments for the reinforced concrete beams Digital Camera (Canon T2i) for DIC/PIV*
Data format	*Processed and Analyzed*
Experimental factors	*Room and Low temperature, thermal gradient, temperature variations, monotonic load, sustained load, 10 load cycles*
Experimental features	*Effects of low temperature on the static behaviour of reinforced concrete beams with temperature differentials Precision and accuracy of DIC/PIV method with temperature variations*
Data source location	*Kingston, Ontario, Canada*
Data accessibility	*Data is within this article*

## Value of the data

• The experimental data on temperature variations, and thermal gradient due to solar radiation on the structural behavior of concrete bridges can be used in “design of concrete structures for temperature”.
• Limited data exists on the use of DIC/PIV in measuring crack widths of concrete structures, however, the presented data in this work will assist the researchers, designers and construction industry to better understand and recognize this state-of-the-art method and replace this cost-effective accurate method for measuring crack widths with conventional methods.
• The experimental data on the accuracy and precision of DIC camera system in measuring strains at different temperatures determined that the strain data that is obtained through this method needs to be calibrated through independent calibration tests.

## 1 Data

The data given in this paper were obtained from testing large-scale conventionally reinforced concrete beams with and without shear reinforcement. The beams had temperature differentials over their depth and were tested at room and low temperature under monotonic load, sustained load and few load cycles (Table 1).

## 2 Experimental design, materials and methods

### 2.1 Beam tests

**Fig. 1:**
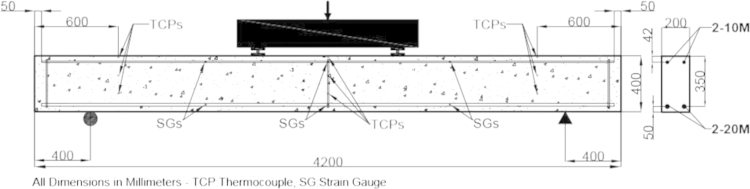
Internal reinforcement, test configuration and instrumentation.

Four large-scale concrete beams, 200 mm×400 mm×4200 mm, with conventional steel reinforcement were constructed. Two 20 Mbar (300 mm^2^) and two 10 Mbar (100 mm^2^) were used as tension and compression reinforcement, respectively. Twenty four 10 M stirrups at 175 mm spacing were used as shear reinforcement for two of the beams. Fig. 1 shows the details of the internal reinforcement, test configuration and instrumentation used in the tested beams.

To simulate solar radiation, heating pads and insulation were placed on the top surface of the beams prior to the start of the tests to create temperature differential over the depth of the beams during the tests.

Two of the simply-supported beams, one with stirrups and one without stirrups, were tested at +15 °C using an electric 900 kN Riehle testing machine and the other counterpart beams were tested using a 500 kN servo-hydraulic actuator in a cold room at −25±2 °C. All of the beams were tested using a four-point bending test setup with a span of 3.4 m and a constant moment zone of 1.0 m as shown in Fig. 1.

**Fig. 2:**
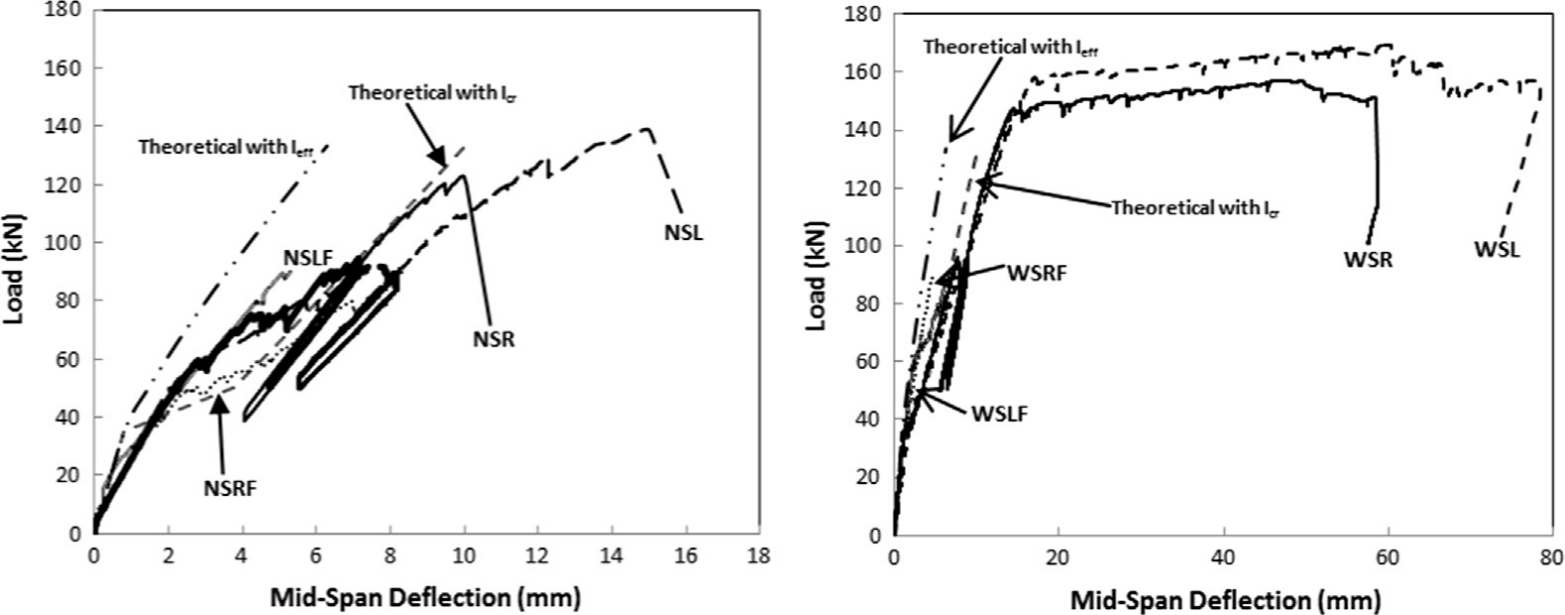
Mid-span deflection for the beams without (left) and with (right) stirrups [1].

Each test consisted of four stages: In the first stage, the beams were incrementally loaded to a service load of 90 kN (in 10 kN increments), then this load (90 kN) was sustained for a period of 48 h during the second stage. The third stage consisted of 10 load cycles between 50 kN and 90 kN, representing variations in service load. Finally, in the fourth stage, the beams were loaded up from 90 kN to failure (Fig. 2).

The load values were measured by the built-in load cells of the electric and hydraulic rams, and deflection values were measured by linear potentiometers (LP) placed at midspan. In addition to the midspan LP, four additional LPs were placed in the middle of the shear spans on opposite sides to monitor possible out-of-plane rotation of the beams.

### 2.2 Measurement of crack widths using DIC

Digital image correlation (DIC) or particle image velocimetry (PIV) is a photomechanical technique. Global and relative displacement and strain variations in structural assessment and full-field vector displacements of small-scale landslides in geotechnical research are the typical examples of the desired information that could be found using this method [2], [3].

In the two-dimensional DIC method, a series of digital images of a deformed object is compared to a digital image of the same object before deformation or the reference image. Using this method, the desired data can be obtained in two stages: recording successive images during the experiment and post-processing the images afterwards using a software package. In the post-processing stage, at first, square subsets or patches are selected from the reference image. To find the displacement vector, a search is performed by the code in a user-specified zone of the deformed image to find the subset with maximum similarity in intensity pattern to the subset׳s signature in the reference image. The difference between the subset location in a post-deformation image and the reference image will be the displacement vector of the subset׳s center which is measured in pixels [3]. In this way, a two-dimensional displacement or strain field is created. In this study, to obtain crack width and strain data, a program called geoPIV, developed by White et al. [4] for monitoring deformations of solids, was employed.

**Fig. 3:**
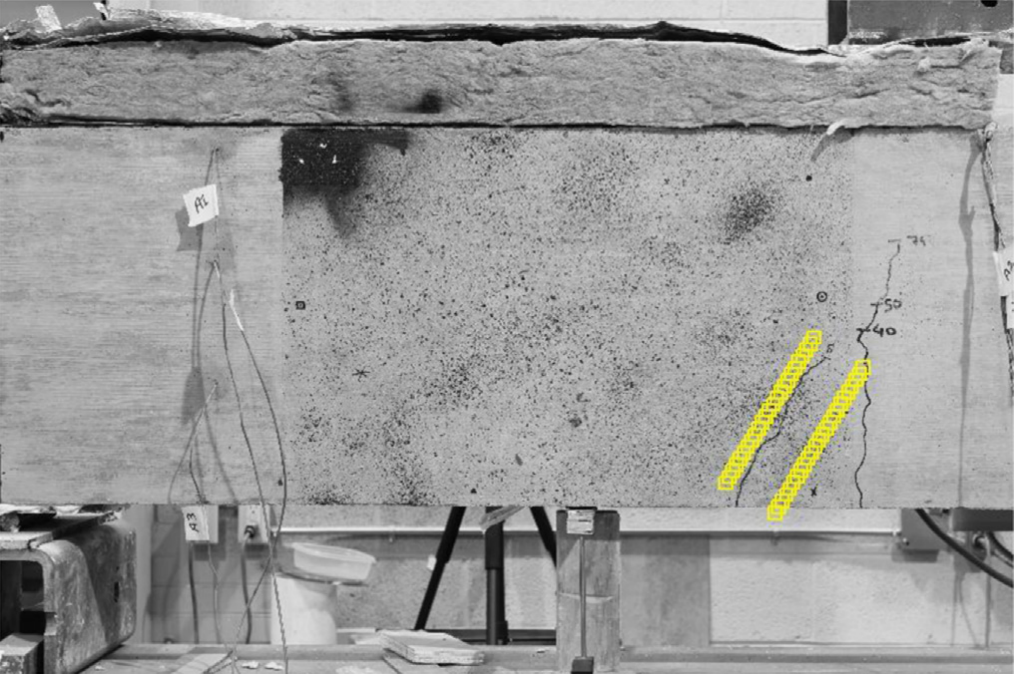
Patches to measure crack widths (post-processing stage of DIC method).

Prior to each test, two digital cameras (Canon EOS Rebel T2i) on tripods were placed at the opposite sides of each beam at the same distance from the beam focusing the central region of one of the shear spans to monitor the shear cracks during the different stages of the each test as can be seen in Fig. 3.

The digital camera itself is affected by the temperature variations [2], [5]; therefore, two independent calibration tests were performed to measure temperature-induced strain errors. Fluctuations in lighting conditions and the quality of the camera affect measurement resolution as well, and digital cameras cannot reproduce the same intensity values of a stationary scene perfectly between multiple exposures, and a slight jitter exists between sequential images [6]. Hence, in order to minimize camera jitter and lighting impacts on the measurements, consecutive images, e.g. five to ten images, should be taken and the average of the these images should be used instead of using a single image [7]. For this reason, five to ten consecutive images were taken at each load stage, then the consecutive images were averaged together using MATLAB to form a single average image at each load step.

In order to measure crack widths, a MATLAB function [3] linked to geoPIV, was used to place columns of patches (64×64 pixels) on either side of the crack in a direction perpendicular to the crack plane as shown in Fig. 3. The two columns of patches act as a ‘virtual strain gauge’ with a 300 pixel gauge length. To measure variations of crack widths along the crack plane, the images of the cracked beam are usually compared to the reference image. However, in this research, in order to reduce error in the measurements, the images were compared to the corresponding image from the previous load stage and then the readings were added up.

### 2.3 DIC calibration

To obtain experimental data on the behavior of the two digital cameras that were used in this study with temperature variations, and to compensate the DIC results for temperature, the response of each DIC camera system at various temperatures was determined using calibration tests.

**Fig. 4:**
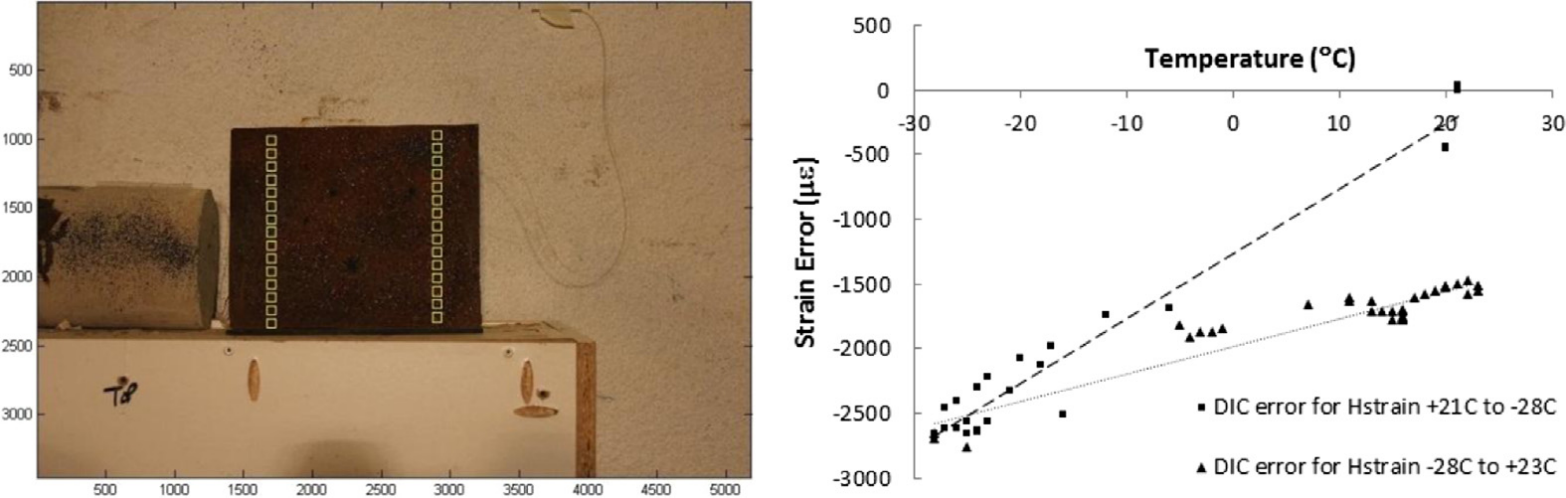
Calibration test setup (left) and temperature-induced error in DIC strain measurements (right).

The two Canon T2i cameras that were used in the beam tests were placed in the cold room and were focused on an unrestrained steel plate that was instrumented with two strain gauges and one thermocouple as shown in Fig. 4. At first, a reference image was taken from the plate at +21 °C, and then the camera timers were set to take photos every two hours while the room temperature was gradually lowered to −25 °C. The room remained at −25 °C for 48 h and then the room temperature was gradually raised back to +24 °C. To determine the strain error (the strain induced into the camera systems due to varying temperature), the strain gauge readings were subtracted from the strains calculated by the DIC method.

## Figures and Tables

**Table 1:** Crack widths obtained from DIC/PIV method [1].

**Beam description**	**Crack widths (mm)**			
Monotonic load (at 90 kN)	48 h Sustained load (90 kN)	10 Load cycles (50–90 kN)	Monotonic load (90 kN to failure)
No shear reinf.-room Temp.	0.14	0.22	0.25	0.28
With shear reinf.-room temp.	0.07	0.10	0.21	0.27
No shear reinf.-low temp	0.12	0.15	0.17	NA
With shear reinf.-low temp	0.05	0.09	0.13	NA
