# Supplementary material for: Experimental data of the static behavior of reinforced concrete beams at room and low temperature
Source: Data Brief. 2016 Apr 16;7:1358–63. doi: 10.1016/j.dib.2016.04.025 (PMC4845156; doi:10.1016/j.dib.2016.04.025)
Supplement: Supplementary file 1 — Supplementary material [file mmc1.docx]

M.Mehdi.Mirzazadeh^1^

Martin Noel^2^

Mark.F.Green^3^

Department of Civil Engineering, Queen’s University,

58 University Ave., Kingston, Ontario, K7L 3N6

**April 5, 2016**

Editor(s) of DIB,

Dear Editor(s),

Attached, please find our manuscript entitled *“EXPERIMENTAL DATA OF THE STATIC BEHAVIOR OF REINFORCED CONCRETE BEAMS AT ROOM AND LOW TEMPERATURE”.*

This article provides data on the static behavior of reinforced concrete at room and low temperature including, strength, ductility, and crack widths of the reinforced concrete. The experimental data on the application of Digital Image Correlation (DIC) or Particle Image Velocimetry (PIV) in measuring crack widths and the accuracy and precision of DIC / PIV method with temperature variations when is used for measuring strains is provided as well.

The authors whose names are listed immediately below certify that they have NO affiliations with or involvement in any organization or entity with any financial interest (such as honoraria; educational grants; participation in speakers’ bureaus; membership, employment, consultancies, stock ownership, or other equity interest; and expert testimony or patent-licensing arrangements), or non-financial interest (such as personal or professional relationships, affiliations, knowledge or beliefs) in the subject matter or materials discussed in this manuscript.

We the undersigned declare that this manuscript is original, has not been published before and is not currently being considered for publication elsewhere.

We wish to confirm that there are no known conflicts of interest associated with this publication and there has been no significant financial support for this work that could have influenced its outcome.

We confirm that the manuscript has been read and approved by all named authors and that there are no other persons who satisfied the criteria for authorship but are not listed.

We further confirm that the order of authors listed in the manuscript has been approved by all of us.

We confirm that we have given due consideration to the protection of intellectual property associated with this work and that there are no impediments to publication, including the timing of publication, with respect to intellectual property. In so doing we confirm that we have followed the regulations of our institutions concerning intellectual property.

We understand that the Corresponding Author is the sole contact for the Editorial process (including Editorial Manager and direct communications with the office). He is responsible for communicating with the other authors about progress, submissions of revisions and final approval of proofs.

We confirm that we have provided a current, correct email address which is accessible by the Corresponding Author and which has been configured to accept email from Elsevier DIB department.

M.Mehdi.Mirzazadeh, PhD candidate

Email:m.mirzazadeh@queensu.ca, Department of Civil Engineering, Queen’s University

58 University Ave., Kingston, Ontario, K7L 3N6

**Signature**: **Date:** April 5, 2016

Martin Noël, Post-doctoral Fellow at Queens (at the time that this work was done)

Department of Civil Engineering, Queen’s University, 58 University Ave., Kingston, Ontario, K7L 3N6

**Signature**: **Date:** April 5, 2016

Mark.F.Green, Professor

Department of Civil Engineering, Queen’s University,

58 University Ave., Kingston, Ontario, K7L 3N6

**Signature**: ***on behalf of Dr.Mark Green*** **Date:** April 5, 2016
